# Colorectal Cancer-Associated Genes Are Associated with Tooth Agenesis and May Have a Role in Tooth Development

**DOI:** 10.1038/s41598-018-21368-z

**Published:** 2018-02-14

**Authors:** Meredith A. Williams, Claudia Biguetti, Miguel Romero-Bustillos, Kanwal Maheshwari, Nuriye Dinckan, Franco Cavalla, Xiaoming Liu, Renato Silva, Sercan Akyalcin, Z. Oya Uyguner, Alexandre R. Vieira, Brad A. Amendt, Walid D. Fakhouri, Ariadne Letra

**Affiliations:** 1 Center for Craniofacial Research, University of Texas Health Science Center School of Dentistry, Houston, 77054 USA; 2 Department of Biological Sciences, University of Sao Paulo Bauru Dental School, Bauru, 17012 Brazil; 3 Iowa Institute for Oral Health, College of Dentistry, University of Iowa, Iowa City, 52242 USA; 4 Department of Medical Genetics, School of Medicine, Istanbul University, Istanbul, 34093 Turkey; 5 Department of Epidemiology and Human Genetics, University of Texas Health Science Center School of Public Health, Houston, 77054 USA; 6 Department of Endodontics, University of Texas Health Science Center School of Dentistry, Houston, 77054 USA; 7 Pediatric Research Center, University of Texas Health Science Center McGovern Medical School, Houston, 77054 USA; 8 Department of Orthodontics, Tufts University, Boston, 02111 USA; 9 Departments of Oral Biology and Pediatric Dentistry, University of Pittsburgh School of Dental Medicine, Pittsburgh, 15229 USA; 10 Craniofacial Anomalies Research Center, Carver College of Medicine, University of Iowa, Iowa City, 52242 USA; 11 Department of Diagnostic and Biomedical Sciences, Sciences University of Texas Health Science Center School of Dentistry, Houston, 77054 USA

## Abstract

Previously reported co-occurrence of colorectal cancer (CRC) and tooth agenesis (TA) and the overlap in disease-associated gene variants suggest involvement of similar molecular pathways. Here, we took an unbiased approach and tested genome-wide significant CRC-associated variants for association with isolated TA. Thirty single nucleotide variants (SNVs) in CRC-predisposing genes/loci were genotyped in a discovery dataset composed of 440 individuals with and without isolated TA. Genome-wide significant associations were found between TA and *ATF1* rs11169552 (P = 4.36 × 10^−10^) and *DUSP*10 rs6687758 (P = 1.25 × 10^−9^), and positive association found with *CASC8* rs10505477 (P = 8.2 × 10^−5^). Additional CRC marker haplotypes were also significantly associated with TA. Genotyping an independent dataset consisting of 52 cases with TA and 427 controls confirmed the association with *CASC8*. Atf1 and Dusp10 expression was detected in the mouse developing teeth from early bud stages to the formation of the complete tooth, suggesting a potential role for these genes and their encoded proteins in tooth development. While their individual contributions in tooth development remain to be elucidated, these genes may be considered candidates to be tested in additional populations.

## Introduction

Colorectal cancer (CRC) is the third most commonly diagnosed cancer in the world and a leading cause of cancer-related deaths^1^. Its etiology is multifactorial and ~33% of all cases are attributed to genetic factors^2^. Tooth agenesis (TA), the congenital absence of one or more permanent teeth, results from disturbances occurring during tooth development, and represents one of the most common craniofacial anomalies in humans^3^. Over a decade ago, Lammi *et al*.^4^ reported that mutations in the tumor suppressor gene *AXIN2* were found co-segregating with colorectal cancer and tooth agenesis in a large multiplex family. Moreover, these authors showed the expression of *AXIN2* in developing mouse teeth and suggested that genes involved in colorectal cancer could also be involved in tooth development^4^.

Tooth development requires a sophisticated series of signaling interactions between the oral epithelium and mesenchyme under strict genetic control by a number of signaling molecules and their downstream signaling pathways^5^. Any alteration of the epithelial-mesenchymal interactions can have deleterious effects on tooth development, and may affect growth, differentiation and pattern formation, or even have systemic effects^6^. TA can occur as part of a syndrome, although it is more frequently found as an isolated trait that may appear sporadically or segregating in families, for which the prevalence ranges between ~2–10% excluding third molars. Based on the number of missing teeth, TA is referred to as hypodontia (up to 5 teeth missing), oligodontia (≥6 teeth missing), or anodontia (all teeth missing), the latter being mostly associated with syndromic TA^3^.

Studies in mice have revealed more than 200 genes involved in tooth development and shown the functional importance of *Msx1, Pax9, Pitx2*, and *Lef1* genes in proper tooth formation and morphogenesis, since the absence of these genes results in arrest of tooth development at the bud stage^7–9^. In humans, the best characterized mutations for both syndromic and nonsyndromic TA involve *MSX1* and *PAX9*^7,10^, with concordance between mouse and human phenotypes. Additional mutations in genes belonging to the canonical Wnt pathway (i.e., *AXIN2, LRP6, WNT*10*A, WNT*10*B*) have also been increasingly implicated in the susceptibility to nonsyndromic TA^11–14^, although mouse models have not yet been able to replicate the human phenotypes. Interestingly, Wnt pathway genes have also been shown to play critical roles in tumorigenesis, particularly in colorectal cancer^4,15–17^.

In addition to epithelial-mesenchymal interactions, cell growth and cell differentiation, the signaling pathways involved in tooth development (i.e., WNT, BMP, SHH, FGF, TGF-β, and NF-kB), also overlap with those implicated in colorectal cancer^6,11^. Recently, genome-wide association studies (GWAS) have uncovered over 50 loci and single nucleotide polymorphisms (SNPs) that are significantly correlated with the susceptibility to CRC, most of which map to genes with established roles in tumorigenesis, or involved in developmental processes such as transcriptional regulation, genome maintenance, and cell growth and differentiation^18–20^. Of note, SNPs in *CDH1, BMP2, BMP4*, and *GREM1* genes, known to have important roles in craniofacial and/or tooth development^6^, have also been reported in association with CRC^6,18^.

Given the previously reported co-occurrence of CRC and TA and the overlap in disease-associated pathways, we assessed the association of genome-wide associated CRC variants with isolated TA. Here, we describe the association of CRC gene polymorphisms with isolated TA and reveal potentially novel candidate genes for TA in humans and mice.

## Results

### GWAS-based association study

A total of 440 unrelated Caucasian individuals, 93 with nonsyndromic TA (29 males, 64 females) and 347 unrelated control individuals without TA or family history of TA (101 males, 246 females) were included in this study. All individuals were examined by a dentist, and the presence of tooth agenesis was determined through clinical and radiographic examinations, and considered if one or more permanent teeth were missing from the oral cavity, excluding third molars. Individuals showing signs of syndromic forms of tooth agenesis (i.e., ectodermal dysplasia features) were excluded. Of the 93 cases with TA, 61 presented with hypodontia while 32 presented with oligodontia. Positive family history of cancer was reported by 28 cases and 39 control individuals and included various cancer types including colorectal cancer (Supplementary Table 1).

We selected 30 CRC-predisposing SNVs with genome-wide significance (5 × 10^−8^) from previously published GWAS^19,21,22^, for genotyping in our case-control dataset (Table 1). For regions where multiple SNPs have been reported, we included the most significantly-associated SNP according to the Genetics and Epidemiology of Colorectal Cancer Consortium (GECCO; https://share.fhcrc.org/sites/gecco/), and prioritized additional SNPs for genotyping based on: (1) location in regulatory or enhancer regions of a given gene, (2) having an effect on motifs in the distal regulatory regions of respective genes, and/or (3) interactions with known genes of biological relevance. Genotypes were generated using Taqman chemistry^23^ and automatic call rate was >98%.

**Table 1:** Details of SNPs investigated in this study.

Locus	SNP	Base Position	Gene	SNP Location	Alleles^a^	MAF CEU^b^
1q41	rs6691170	221872104	*DUSP10*	downstream	G/T	0.32
	rs6687758	221991606		downstream	A/G	0.25
2q32.3	rs11903757	191722478	—	intergenic	T/C	0.12
3q26.2	rs10936599	169774313	*MYNN*	synonymous coding	C/T	0.26
5q31.1	rs647161	135163402	*C5orf66*	intron	C/A	0.36
6p21	rs1321311	36655123	—	intergenic	T/G	0.29
8q24	rs10505477	127395198	*CASC8*	intron	T/C	0.45
	rs6983267	127401060		intron	G/T	0.42
	rs7014346	127412547		intron	G/A	0.37
9p24	rs719725	6365683	—	intergenic	A/C	0.39
10p14	rs10795668	8659256	—	intergenic	G/A	0.27
	rs1665650	116727589	*HSPA12A*	intron	A/G	0.28
11q13.4	rs3824999	74634505	*POLD3*	intron	A/C	0.46
11q23	rs3802842	111300984	*COLCA2*	intron	C/A	0.29
12p13.32	rs10774214	4259186	*CCND2*	intron	C/T	0.39
	rs3217810	4279105		intron	C/T	0.07
	rs3217901	4296223		intron	A/G	0.39
12q13.13	rs7136702	50486433	*DIP2B*	upstream	T/C	0.48
	rs11169552	50761880	*ATF1*	near gene 5'	C/T	0.27
12q24.21	rs59336	114678547	*TBX3*	intron	A/T	0.47
14q22.2	rs4444235	53944201	*BMP4*	downstream	T/C	0.50
	rs1957636	54093300	—	intergenic	A/G	0.50
15q13	rs4779584	32702555	*SCG5*	downstream	T/C	0.24
	rs11632715	32712046	*GREM1*	upstream	A/G	0.43
16q22.1	rs9929218	68787043	*CDH1*	intron	G/A	0.26
18q21	rs4939827	48927093	*SMAD7*	intron	T/C	0.47
20p12.3	rs961253	6423634	—	intergenic	A/C	0.29
	rs4813802^c^	6718948		intergenic	T/G	0.22
20q13.33	rs4925386^c^	62345988	*LAMA5*	intron	C/T	0.20
Xp22.2	rs5934683^c^	9783434	*SHROOM2*	upstream	T/C	0.50

^a^Reference allele listed first.

^b^MAF, minor allele frequency in CEU population (from NCBI dbSNP database).

^c^Out of Hardy-Weinberg equilibrium and excluded from further analyses.

Genome-wide significant associations were found between TA and individual SNPs on 1q41 and 12q13 (Table 2). At the 1q41 locus, rs6687758 is located downstream of the dual specificity phosphatase 10 (*DUSP*10*)* gene, and allelic (P = 2 × 10^−9^) and genotypic (P = 1.25 × 10^−9^) associations were found between this SNP and TA, particularly under a recessive model (P = 1.27 × 10^−9^). At the 12q13.1 locus, rs11169552 is located 2Kb upstream of the activating transcription factor 1 (*ATF1)* gene promoter, for which genome-wide significant genotypic (P = 4.36 × 10^−10^) and positive allelic associations (P = 1.87 × 10^−7^) were also found (Table 2). Positive association was also found between intronic variants in the cancer susceptibility 8 (*CASC8)* gene on chromosome 8q24 and TA for SNP rs10505477 genotypes and alleles (P = 8.16 × 10^−5^ and P = 1.7 × 10^−5^, respectively) and rs7014346 (P = 0.0005 for genotype under a recessive model) (Table 2). When stratifying analyses by cases with hypodontia or oligodontia phenotypes, these associations remained significant for both phenotypes, despite the smaller number of oligodontia cases (P ≤ 0.002, data not shown). To confirm our findings, we genotyped the associated SNPs on an independent dataset composed of 52 cases with tooth agenesis and 427 gender- and ethnicity-matched unrelated controls, and found significant association between *CASC8* rs10505477 (P = 0.006 for genotype and P = 0.008 for allele) and TA, particularly under a dominant model (P = 0.001).

**Table 2:** Summary of association results under different genetic test models.

Gene	SNP Id.	MAF CEU^a^	MAF Case	MAF Control	Test	Alleles	Frequency Cases	Frequency Controls	P-value^b^
*ATF1*	rs11169552				Genotype	CC/CT/TT	38/31/24	195/129/14	**4.36 × 10** ^−**10**^
		0.27(T)	0.42(T)	0.24(T)	Allele	C/T	107/79	519/157	*1.85* × *10*^−*7*^
					Dominant	CC × CT + TT	38/55	195/143	0.004
					Recessive	CC + CT × TT	69/24	324/14	**6.79 × 10** ^−**11**^
*DUSP10*	rs6687758				Genotype	AA/AG/GG	37/34/22	217/112/14	**1.25 × 10** ^−**9**^
		0.25(G)	0.42(G)	0.20(G)	Allele	A/G	108/78	546/140	**2.09 × 10** ^−**9**^
					Dominant	AA × AG + GG	37/56	217/126	*5.09* × *10*^−*5*^
					Recessive	AA + AG × GG	71/22	329/14	**1.27** × **10**^−**9**^
*CASC8*	rs10505477				Genotype	TT/TC/CC	16/45/32	119/167/56	*8.16* × *10*^−*5*^
		0.45(C)	0.41(T)	0.41(C)	Allele	T/C	77/109	405/279	*1.69* × *10*^−*5*^
					Dominant	TT × TC + CC	16/77	119/223	*0.001*
					Recessive	TT + TC × CC	61/32	286/56	*0.0001*
	rs7014346				Genotype	GG/GA/AA	31/38/24	145/154/39	*0.002*
		0.37(A)	0.46(A)	0.34(A)	Allele	G/A	100/86	444/232	0.003
					Dominant	GG × GA + AA	31/62	145/193	0.09
					Recessive	GG + GA × AA	69/24	299/39	*0.0005*

MAF, minor allele frequency.

^a^Reference allele.

^b^Fisher Exact test, denotes genome-wide significant association if P ≤ 5 × 10^−8^ (bolded) and positive association under Bonferroni correction if P ≤ 0.002 (italicized).

Significant haplotype associations were also observed and included the individually associated SNPs. The strongest association was observed for the combination of *CASC8* rs10505477, rs7013436, and rs6983267 alleles (CAG haplotype, P = 5 × 10^−13^; CAT haplotype, P = 4 × 10^−9^), followed by *DUSP10* rs6687758 and rs6691170 (GG haplotype, P = 7.32 × 10^−10^), and *ATF1* rs7136702 and rs11169552 (TC haplotype, P = 4.8 × 10^−8^) (Supplementary Table 2). Interestingly, when considering TA in the presence of family history of cancer, positive association was found for carriers of a TT genotype (thus two copies of the minor allele T) in *ATF1* rs11169552 (P = 0.00004), and for carriers of a GG genotype in *DUSP10* rs6687758 (P = 0.003) (Supplementary Table 3).

### Functional annotation of associated variants

As a first step into assessing the relevance of the associated genes in tooth agenesis phenotypes, we annotated the associated variants using the dbSNP^24^ and the UCSC Genome Browser^25^ databases and to predict gene and variant function. Our analyses showed that the associated variants, although common, are predicted to have effects on chromatin structure and RNA polymerase activity and on transcription of their respective genes. *ATF1* rs11169552 is located in the gene promoter in a region showing high DNase I hypersensitivity clusters and potential location of regulatory elements (enhancers, silencers, insulators, and locus control region) (Supplementary Figure 1). Further, *ATF1* rs11169552 is predicted to harbor a binding site for *POLR2A*, essential for RNA polymerase activity and DNA transcription. *DUSP10* rs6687758 is located in a DNAse I hypersensitivity cluster with binding motif for TBP (TATA-box binding protein) (Supplementary Figure 2). *CASC8* rs10505477 appears to harbor a putative binding motif to NK2–5, in a region of enriched H3K27Ac histone marks (Supplementary Figure 3). These findings suggest that these variants are likely to have functional consequences on gene expression.

We attempted to presume the effects of the associated SNPs on expression of their respective genes by searching for evidence of eQTLs at the respective loci, however no data was available in relevant oral/dental tissues to yield conclusive evidence (data not shown).

### Expression analyses

Since no information was available from public databases regarding the expression of *ATF1, DUSP10 and CASC8* during tooth development, as a second step into determining the relevance of these genes in tooth development, we sought to assess whether their transcripts and/or encoded proteins were expressed in the oral and dental tissues of developing mouse embryos.

**Figure 1:**
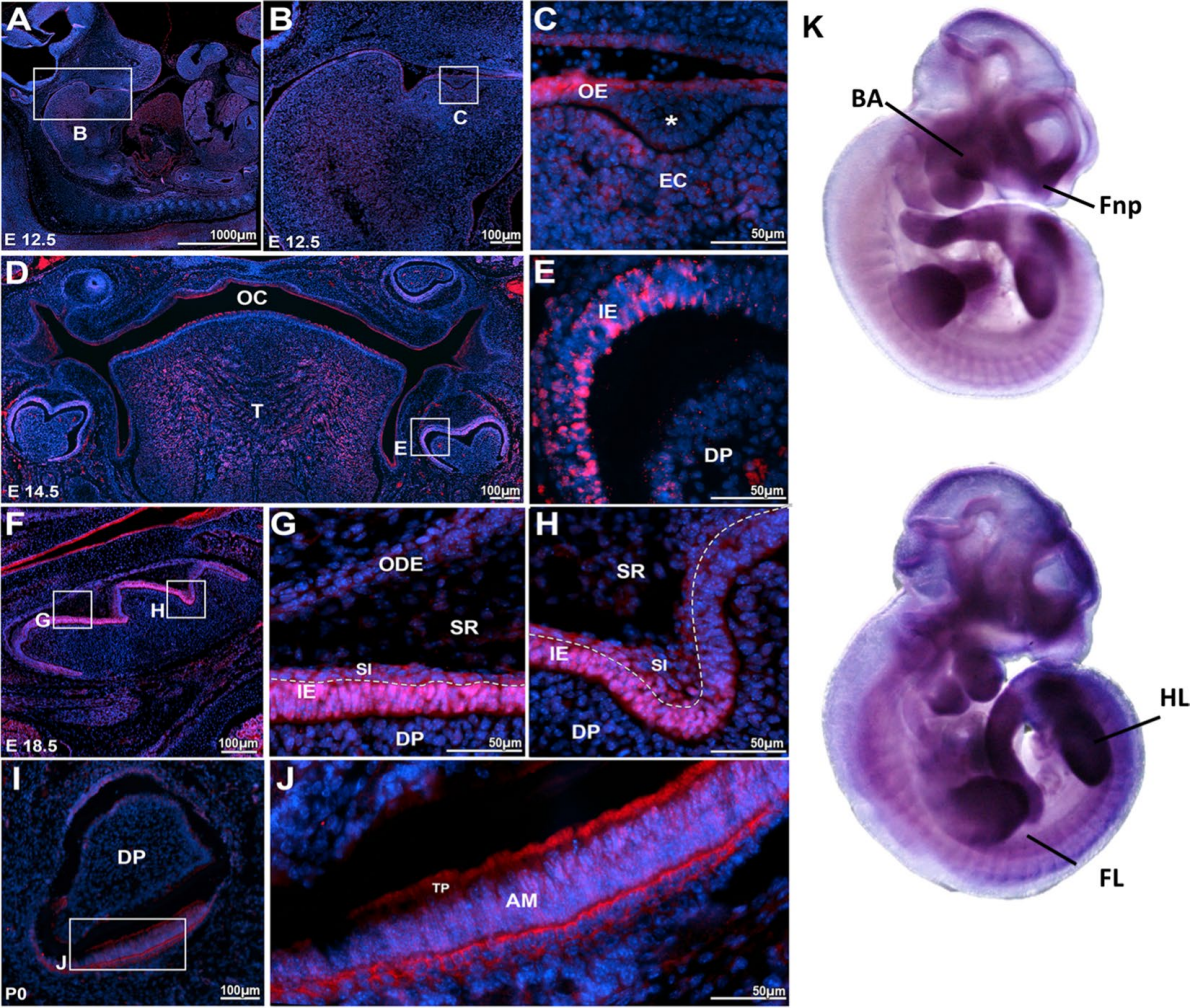
Atf1 is expressed in mouse developing teeth. (**A**,**B**) Sagittal section of mouse embryo at E12.5. **(C)** Proliferation (*) of the oral epithelium (OE) into the ectomesenchyma, (EM) consistent with the formation of a molar tooth bud at early bud stage. ATF1 expression was detected the oral epithelium, as well as in the condensed ectomesenchymal cells. **(D)** Panoramic photomicrography showing the mouse oral cavity (OC) with tooth germs at early bell stage (E14.5). (**E**) ATF1 expression was noted in the inner enamel/dental epithelium (IE) and dental papilla (DP). (**F**–**H**) At E16.5 (Appendix Figure 1) and E18.5, marked ATF1 expression was observed in the inner enamel epithelium (IE) and in the stratum intermedium (SI), with sparse expression noted in the stellate reticulum (SR) and outer dental (ODE) epithelium. (**I**–**J**) In incisor teeth at P0, ATF1 expression was particularly evident in the polarized layer of ameloblasts, and in the Tomes’ processes (TP). (**K**) *Atf1* mRNA detected by whole mount *in situ* hybridization with digoxigenin-labeled antisense RNA followed by alkaline phosphatase-coupled antibody against digoxigenin in C57BL mice at embryonic day 10.5. Strong expression is noted in the mesenchyme of frontonasal prominences, branchial arches and limbs (Obtained from MGI Gene Expression Database. Original source: Gray *et al*. Mouse Brain Organization Revealed Through Direct Genome-Scale TF Expression Analysis. Science. 2004 24;306(5705): 2255–2257). Secondary antibody goat anti-rabbit-Alexa 555 for detection of ATF1 and DAPI for nuclear staining. OE = Oral ephitelium; EM = ectomesenchymal cells; OC = oral cavity; T = tongue; IE = inner epithelium; DP = dental papilla; SI = stratum intermedium; SR = stellate reticulum; ODE outer dental ephithelium; AM = ameloblasts; TP = Tomes’ processes; BA = branchial arches; Fnp = frontonasal processes; HL = hind limbs; FL = forward limbs.

**Figure 2:**
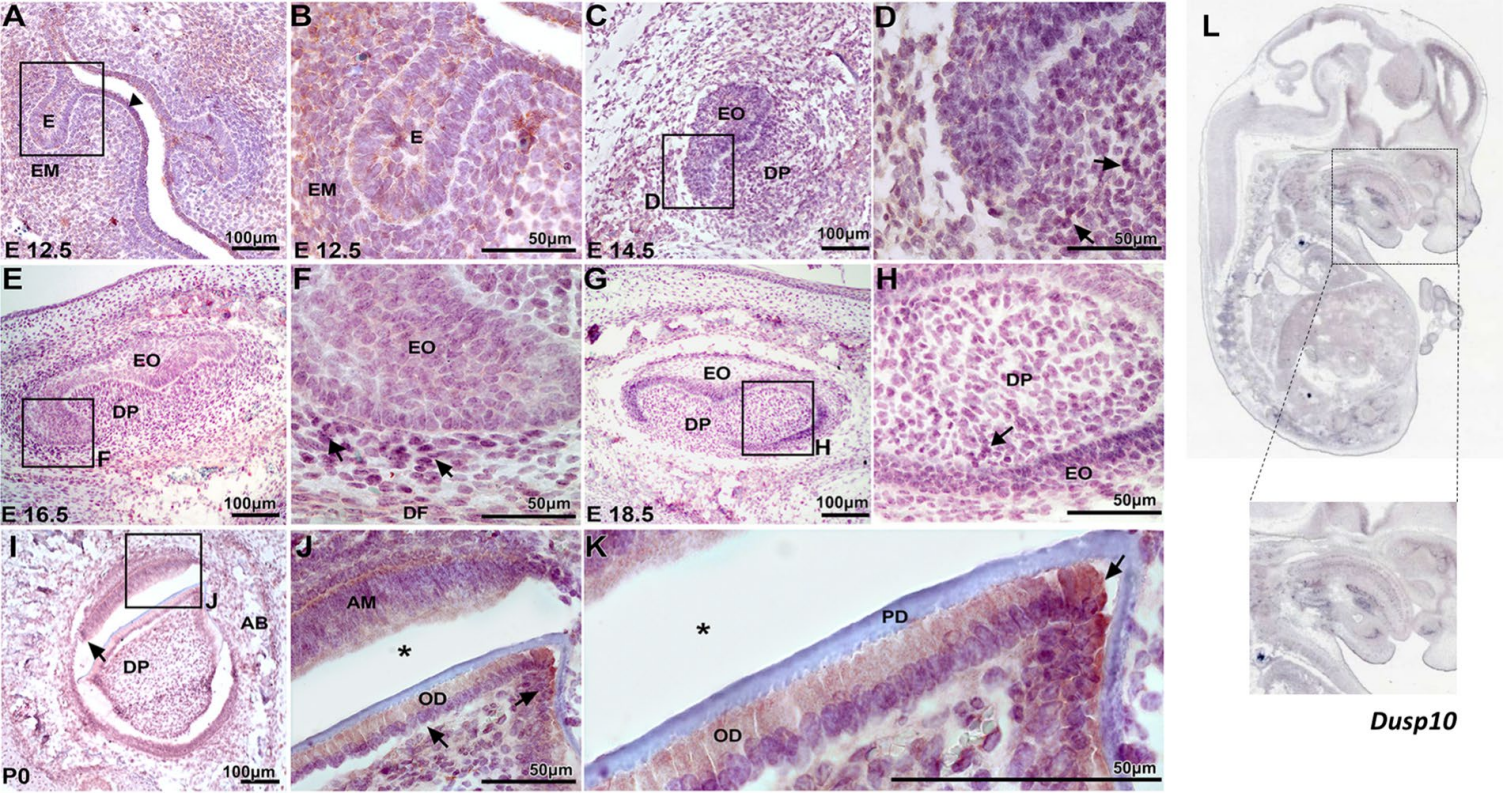
Dusp10 expression in mouse developing teeth. (**A**–**B**) At E12.5, there is a proliferation of the dental lamina surrounded by a condensation of the underlying ectomesenchymal cells (EM) compatible with a bud stage of tooth development. Dusp10 expression was noted in the epithelial cells from oral epithelium (arrowhead), as well as in the condensed ectomesenchymal cells surrounding the epithelial proliferation (**E**). **(C**,**D**) At E14.5, expression was noted detected in the enamel organ (EO) and dental papillae (DP) (arrows). **(E,F**) At bell stage E16.5, Dusp10 positive staining is observed in the enamel organ, dental papillae and dental follicle (DF) cells (arrows). **(G,H)** At E18.5, the tooth germ demonstrates morphodifferentiation compatible with a bell stage. The enamel organ (EO) cells, ectomesenchymal cells of the dental papillae (DP) and the dental follicle cells show homogenous nuclear immune staining. **(I**–**K)** At P0, the incisor tooth germ is surrounded by alveolar bone (AB) and shows morphology compatible with a late bell stage or crown stage, such as ameloblasts (AM), odontoblasts (OD) and pre-dentin (PD) deposition. Dusp10 expression was observed in the preameloblasts and subjacent odontoblast layer (arrows). **(L)** D*usp10* mRNA detected by whole mount *in situ* hybridization in C57BL/6 mouse at E14.5. Positive expression is noted in the craniofacial region and oral cavity, particularly in the mouth and tongue. (Obtained from MGI Gene Expression Database. Original source: Hoffman *et al*. Genome Biol 2008;9(6):R99). An artifact (*) separated the preameloblasts from the pre-dentin and odontoblastic layer. DAB chromogen and counterstaining with Mayer’s Hematoxylin. OE = Oral ephitelium; EM = ectomesenchymal cells; EO = enamel organ; DP = dental papilla; AM = ameloblasts; AB = alveolar bone.

We performed immunofluorescence and immunohistochemistry procedures to detect the localization of Atf1 and Dusp10, respectively, during murine tooth development stages. At E12.5, Atf1 expression was detected in the oral epithelial cells and in the subjacent condensed ectomesenchymal cells (Fig. 1A,C). Later, at E14.5, Atf1 expression was evident in the oral and tongue epithelia, and underlying mesenchyme, in the inner dental epithelium (IE) and in few scattered cells of the dental papilla (DP) (Fig. 1D–E). At E16.5 (Supplementary Figure 4), and E18.5, Atf1 was markedly expressed in the inner epithelium and the stratum intermedium. Reduced expression was also noted in the cells from the stratum intermedium, stellate reticulum and outer dental epithelium (Fig. 1F,H). At P0, Atf1 expression shifted to the cytoplasm of the polarized layer of ameloblasts, in a perinuclear pattern in the ameloblast bodies, as well as in the Tomes’ processes (Fig. 1I,J). *Atf1* mRNA expression had been shown to be expressed in mouse embryos as early as E10.5, with strong expression noted in the mesenchyme of frontonasal prominences, branchial arches and limbs^26^ (Fig. 1K). Dusp10 expression was detected at a site of proliferation of the dental placode at E12.5, surrounded by a condensation of the underlying ectomesenchymal cells compatible with a bud stage of tooth development. Expression was evident in the epithelial cells from the oral epithelium, as well as in the condensed ectomesenchymal cells surrounding the epithelial proliferation (Fig. 2A,B). At E14.5, nuclear and cytoplasmic staining was observed on the cells from the enamel organ and dental papillae (Fig. 2C,D). At bell stage E16.5, positive staining was observed in the enamel organ, dental papillae and dental follicle cells (Fig. 2E,F). At 18.5, Dusp10 expression was noted in the enamel organ, in the ectomesenchymal cells of the dental papillae and the dental follicle cells (Fig. 2G,H). Later, at P0, Dusp10 expression was localized to the preameloblasts, odontoblasts and pre-dentin (Fig. 2I,K, Supplementary Figure 5). Marked expression was noted in the nuclei and cytoplasms of preameloblasts, as well as in the subjacent odontoblast layer (Fig. 2K). *Dusp10* mRNA expression was observed in the mouse craniofacial region and oral cavity at E14.5, particularly in the mouth and tongue^27^. (Fig. 2L). These data suggested that Atf1 and Dusp10 are present throughout tooth development stages in mouse embryos and thus likely to have a role in tooth development. Therefore, variations that impact the function of ATF1 and DUSP10 in humans are likely to play a contributory role in tooth agenesis.

*CASC8* has only recently been annotated in the human genome, and not yet in the mouse genome, and thus limited our analysis to evaluate its expression in relevant tissues. Using information available in public databases, we found that *CASC8* mRNA is expressed in many tissues including brain, skin, minor salivary gland, colon and esophagus. We then performed RT-PCR analysis of *CASC8* expression in stem cells of the apical papilla (SCAP), a dental-derived stem cell line^28^, however, no expression was detected.

## Discussion

Over a decade ago, Lammi *et al*.^4^ identified *AXIN2* as a novel gene involved in tooth development after finding mutations in this gene to be co-segregating with CRC and TA in a large multiplex family. Moreover, these authors showed the expression of *Axin2* in developing mouse teeth and showed for the first time that a gene involved in CRC could also be involved in tooth development. Here, we describe the findings of CRC-predisposing genes with potential roles in tooth development and as new candidate genes for isolated TA.

From the thirty CRC-predisposing variants assessed, our association analyses revealed genome-wide significant associations between TA and loci on 1q41 and 12q13, containing *DUSP10* (dual specificity phosphatase 10) and *ATF1* (activating transcription factor 1) genes, respectively. Meanwhile, positive associations (although not strong to reach the genome-wide significance threshold level) were also found between *CASC8* (cancer susceptibility candidate 8) and TA. These genes had not yet been described to have a role in tooth development and prompted investigations regarding their biological relevance to the TA phenotype. Intriguingly, no associations were found between TA and *CDH1, BMP2, BMP4*, or *GREM1* genes, known to have important roles in craniofacial and/or tooth development^6^, and which have also been reported in association with CRC^6,18^.

*ATF1* belongs to the ATF/CREB family of transcription factors, which binds to the consensus ATF/CRE site ‘TGACGTCA’ and regulates the transcription of target genes to participate in various cellular processes^29^. Previous reports showed that the function of *ATF1* appears to depend on the cellular and genetic context to play an important role in tumor progression in a tumor-specific manner^30^. ATF1 was found to be over-expressed in several cancer types including lymphoma, melanoma and nasopharyngeal carcinoma, and functioned as a tumor promoter both *in vitro* and *in vivo*^31–34^. In contrast, disruption of ATF1 activity suppressed its tumorigenicity and metastatic potential^33^. However, the exact role of *ATF1* during craniofacial development is still largely unknown. Loss of *Atf1* in mice did not cause any obvious phenotypic abnormalities although led to increased programmed cell death^35^.

DUSP10 is a dual specificity phosphatase (DUSP) that negatively regulates the activation of the mitogen-activated protein kinase (MAPK) gene family, which includes the extracellular-regulated kinases (ERKs) and the stress-activated protein kinases p38 and c-Jun NH_2_-terminal kinase (JNK). Activation of MAPK by various stimuli including growth factors, cytokines, or stress conditions, regulates major cellular responses such as proliferation, differentiation, survival, migration or production of soluble factors^36^. *Dusp10* has been regarded as an important regulator of tumorigenesis in animal models^37^, and loss of *Dusp10* in mice caused enhanced immune and inflammatory responses although effects on skeletal phenotype have not been described^38^. Nonetheless, other DUSPs have been found to play critical roles in development. Loss of function studies revealed that Dusp4 is essential for early development and endoderm specification in the zebrafish^39^, whereas Dusp5 was described to control angioblast populations in the lateral plate mesoderm^40^.

*CASC8* is a long noncoding RNA (lncRNA), located in the gene desert region on 8q24.21. LncRNA is a new class of transcripts that are involved in multiple cellular functions including the regulation of expression of multiple genes. Studies have shown that SNPs in lncRNAs may affect the biological processes of messenger RNA conformation, and results in the modification of its interacting partners. Interestingly, various cancer types, including CRC, prostate, breast and gastric cancer, have been reported in association with *CASC8*^22,41^, possibly through its function as a lncRNA.

Variants in *ATF1, DUSP10 and CASC8* have been associated with increased risk of CRC in different GWAS with multiple populations^19,21,22^, although functional studies addressing the potential effects of these variants on gene function and/or disease mechanisms are scarce. While the biological function of the associated *ATF1, DUSP10*, and *CASC8* variants is yet to be determined, our bioinformatics analyses revealed that all three variants are predicted to be within DNAse I hypersensitivity clusters, or in enhancer histone marks, with likely functional effects on gene expression. Moreover, *ATF1* rs11169552 is predicted to harbor a binding site for *POLR2A*, essential for RNA polymerase activity and DNA transcription. Another regulatory variant in *ATF1* (rs11169571) was shown to increase breast/ovarian cancer risk through modifying miRNA binding^42,43^. Interestingly, this variant is in linkage disequilibrium with *ATF1* rs11169552, associated in the present study, and could be transmitting the same genetic information as well as functional potential. *DUSP10* rs6687758 is predicted to have a putative binding motif for TBP (TATA-box binding protein), which controls the transcription machinery. *CASC8* rs10505477 has a putative binding motif to NK2-5, a homeobox-containing transcription factor with roles in embryonic development. These findings suggest that these genes are active in early cell processes being important in both embryogenesis and tumorigenesis.

The relevance of *ATF1, DUSP10* and *CASC8* in tooth development is unknown. Therefore, we assessed the expression of these genes or their encoded proteins in relevant tissues or cell lines. During mouse embryonic development, *Atf1* mRNA was detected at embryonic day 10.5, with strong expression noted in the mesenchyme of the frontonasal prominences, branchial arches and limbs^26^. Our findings showed that Atf1 is expressed throughout mouse tooth development stages, in the oral epithelial cells and subjacent condensed ectomesenchymal cells during the early bud stage of tooth development, then shifting to the inner dental epithelium and dental papilla, and finally in the ameloblasts at the final stages of tooth development. *Dusp10* mRNA expression was also observed in the mouse craniofacial region and oral cavity at E14.5, particularly in the mouth and tongue^27^. In our study, the expression of Dusp10, albeit weak, was also noted in the proliferating dental tissues at the early stages of tooth development. At later stages, Dusp10 expression was evident in the enamel organ, pre-ameloblasts and odontoblastic layer. Expression of *CASC8* mRNA has been observed in the brain, skin, minor salivary gland, colon and esophagus. However, no expression of *CASC8* was detected in our study with human dental stem cells of the apical papilla (SCAP). Since *CASC8* has only recently been annotated in the human genome (and not yet annotated in mice), we were limited in the choice of relevant tissues to assess its expression thus hampering our interpretation of the role of this gene in tooth development. Of note, the observed association between TA and the *CASC8* locus on chromosome 8q24.21 raises intriguing questions as this locus has been associated with increased risk of several malignancies including CRC^22,41^ and also cleft lip/palate^44,45^, for which an expanded phenotype including tooth agenesis has been proposed^46^. Furthermore, the associated *CASC8* rs10505477, is located at approximately 250–300 Kb away from the transcription start site of *cMYC*, a gene well-known for its reprogramming capacity and as a proto-oncogene. Enhancer elements of cMYC are known to be up to 500 Mb away from the gene start site, therefore it is possible that the *CASC8* SNP may be in a LD block associated with cMYC enhancer elements and drive the association findings^47^. The presence of Myc has been detected in dental pulp cells, dental follicle cells, ameloblasts and odontoblasts, and could be related to cell reprogramming or differentiation during tooth development^48^.

Among the limitations of this study is the limited availability of individual cancer history or family history of cancer. Since most of our patients with TA are relatively young, our individual patient tumor/cancer history was low (3 patients). In addition, the majority of the TA patients’ parents and/or immediate family members are also younger than the ages in which preventive cancer screening begins. Regarding family history of cancer, in some instances, patients were unsure of cancer status in the family and thus their responses were not accounted for. We considered positive family history of cancer when the individual could name the relative that was affected, and despite various cancer types (including colorectal cancer) were reported for relatives of TA patients, the number of cases was considered small to warrant conclusive genotype-phenotype comparisons between cases and controls. It is also possible that the genetic variants associated with TA in the present study may be in linkage disequilibrium with a more distant causal variant, and acting as surrogate markers for the condition. Additional fine-mapping around the associated loci may provide additional insights into the association signal. Lastly, we cannot rule out that additional variants in other genes may also be contributing to the TA phenotype observed. Similar to other genetic conditions, the hypothesis of multilocus genomic variation^49^ was proposed for some families in which a single genetic variant could not explain the TA phenotypes^13^. In this context, the association observed with the CRC-predisposing genes may represent a direct or indirect role in the background of additional causal genes.

In summary, our findings provide evidence that novel genes and gene pathways may be yet unknown for a role in tooth development, and unbiased genetic studies have the potential to identify the full spectrum of candidate genes and variants associated with isolated TA. While the exact contributions of *ATF1, DUSP10* and *CASC8* in TA remain to be further elucidated, our findings further support that genes involved in colorectal cancer may also be involved in tooth development and provide additional insights into deciphering the complex etiology of the condition.

## Methods

### Study Population

This study was approved by the University of Texas Health Science Center at Houston Committee for the Protection of Human Subjects and the University of Pittsburgh Institutional Review Board, and all methods were performed in accordance with the relevant guidelines and regulations. Unrelated individuals with and without tooth agenesis were invited to participate in the study when presenting for treatment at the University of Texas Health Science Center at Houston School of Dentistry clinics. Written informed consent was obtained from all participating individuals and/or from their parents or guardians. Clinical and demographic information, and saliva samples were collected from all individuals. When known, individual and familial history of cancer was also recorded. All individuals were examined by a dentist, and the presence of tooth agenesis was determined through clinical and radiographic examinations, and considered if one or more permanent teeth were missing from the oral cavity, excluding third molars. Differential diagnosis of tooth agenesis from other potential causes of tooth loss (e.g. caries, periodontal disease, trauma) was ensured through comprehensive evaluation of patients’ electronic dental records and conversation with the patient and/or legal guardian about previous tooth extractions and history of trauma. Individuals showing signs of syndromic forms of tooth agenesis (i.e., ectodermal dysplasia features) were excluded.

To avoid potential effects of population stratification in genetic studies, only individuals with self-reported Caucasian ethnicity (up to 2 generations) were included in the study. A total of 440 unrelated individuals, 93 with nonsyndromic TA (29 males, 64 females) and 347 ethnicity-matched, control individuals without TA or family history of TA (101 males, 246 females) were included in this study. No selection regarding gender was used; the female to male ratio observed in this study is similar to the overall gender prevalence reported in the literature.

Of the 93 cases with TA, 61 presented with hypodontia while 32 presented with oligodontia. Positive family history of cancer was reported by 28 cases and 39 control individuals. Details of known cancer types reported are available in Supplementary Table 1.

### Selection of CRC-Associated Variants and Genotyping

We selected 30 CRC-risk-associated SNPs that reached genome-wide significance (5 × 10–8) in previously published GWAS^19,21,22^ to genotype in this study (Table 2). We prioritized the SNPs to investigate in our study, based on the population that they had been investigated to more closely match our study population, and genome-wide significant P-values. For regions where multiple SNPs have been reported, we included the most significantly-associated SNP according to the Genetics and Epidemiology of Colorectal Cancer Consortium (GECCO; https://share.fhcrc.org/sites/gecco/), and prioritized additional SNPs for genotyping based on: (1) location in regulatory or enhancer regions of a given gene, (2) having an effect on motifs in the distal regulatory regions of respective genes, and/or (3) interactions with known genes of biological relevance.

Genotyping was performed using Taqman chemistry^23^ in 5-μL final reaction volumes in a ViiA7 Sequence Detection System (Applied Biosystems, Foster City, CA). Results were analyzed using EDS v.1.2.3 software (Applied Biosystems). For quality control of genotyping reactions, we used a non-template reaction as negative control and a DNA sample of known genotype as positive control. Genotyping was performed in duplicates and genotypes that did not have a 95% pass rate were repeated.

### Data analyses

Power calculations were performed using Genetic Power Calculator (http://zzz.bwh.harvard.edu/gpc/) and indicate that the study sample size provided approximately 86% power to detect an association with an alpha of 0.05, if the markers selected are in linkage disequilibrium with the causal factor (D′ = 0.80) and their frequencies are around 80%. Data analysis was performed using PLINK version 1.06^50^. Hardy-Weinberg equilibrium was calculated for cases and controls, and SNPs showing evidence of deviation in controls were excluded from further analyses. Differences in allele and genotype frequencies for each polymorphism between cases and controls were compared using chi-square and Fisher Exact tests. We corrected for multiple testing using the Bonferroni method, and the significance level was set considering the number of tests (n = 30) to give a corrected P-value (α = 0.002). Haplotype analyses were performed using the ‘haplotype-based case-control association test’ as implemented in PLINK. Regression analyses were performed using EpiInfo v.7.1 (https://www.cdc.gov/epiinfo/) to identify potential preferential associations between specific SNP genotypes with tooth agenesis adjusted for family history of cancer and gender.

### Bioinformatic analyses of SNP function

Annotation of the newly identified loci was performed using the dbNSFP^24^ and FuncPred (https://snpinfo.niehs.nih.gov/snpinfo/snpfunc.html) databases. Information on the function of the associated SNPs regarding splicing regulation, stop codon, Polyphen predictions, transcription factor binding site predictions, miRNA binding site prediction regulatory potential score, conservation score, and nearby genes, were recorded. The presence of expression quantitative trait loci for each of the associated SNPs was assessed using the GTEx portal (gtexportal.org).

### Immunofluorescence and immunohistochemistry

The expression of Atf1 and Dusp10 proteins was evaluated using immunofluorescence and immunohistochemistry in developing mouse tooth sections, respectively. Wild type C57BL/6 mouse embryos at embryonic days (E) 12.5, 14.5, 16.5, 18.5 and postnatal day 0 (P0) were harvested, fixed in 4% paraformaldehyde and embedded in paraffin for sectioning at 7μm of thickness. Sections were deparaffinized in xylene solution, rehydrated in gradative alcohol baths and rinsed with deionized H2O at room temperature. For antigen retrieval, sections were immersed in sodium citrate buffer (10 mM, pH 6) at 100 °C for 30 minutes and allowed to cool down for 30 min at room temperature. To avoid nonspecific binding of the antibodies, sections were immersed in blocking solution (1% bovine serum albumin and 10% goat serum diluted inPBS) and incubated with AffiniPure Fab fragment goat anti-mouse (Jackson ImmunoResearch Laboratories, PA, USA), for 1 hour at room temperature. For Dusp10, an additional blocking step consisted of incubating sections in 2.5% normal horse serum (Vector Laboratories, CA, USA) for 30 min at room temperature. Sections were then washed and incubated with either Atf1 (rabbit polyclonal anti-mouse Atf1; ab189311, Abcam, MA, USA), or Dusp10 (rabbit polyclonal anti-mouse Dusp10; ab71309, Abcam, MA, USA) primary antibodies at 4 °C overnight. For Atf1, sections were then washed and incubated with Alexa Fluor® 555 (goat anti-rabbit secondary antibody, A-21428, ThermoFisher Scientific,MA, USA) for 2 hours in a dark chamber at room temperature. Lastly, sections were washed (3 × 10′), counterstained with DAPI (Invitrogen) in a dark chamber for 10 min, and mounted with ProLong Gold AntifadeReagent (Invitrogen). For Dusp10, sections were incubated with biotinylated horse anti-rabbit IgG (BP-1100, Vector Laboratories, CA, USA) for 30 minutes at room temperature, washed and incubated with Streptavidin/Peroxidase complex (PK7800, Vector Laboratories, CA, USA) for 5 minutes and washed. Sections were then incubated in 3,3′-Diaminobenzidine (ImPACT DAB, Vector Laboratories, CA, USA) chromogen for 2 minutes, washed, and counterstained with Mayer’s Hematoxylin. Lastly, sections were washed, dehydrated and mounted. Imaging was performed after 48 hours in a Nikon Eclipse Ni-U upright fluorescence microscope (Nikon, Germany) equipped with a Zyla 5.5 sCMOS camera (Andor).

### Reverse transcriptase-PCR (RT-PCR)

Total RNA was extracted from SCAP by using the RNeasy kit (Qiagen Inc, Valencia, California, USA). RNA sample integrity was checked by analyzing 1 μg of total RNA on 2100 Bioanalyzer (Agilent Technologies, Santa Clara, California, USA). After RNA extraction, complementary DNA was synthesized by using 3 μg of RNA through a reverse transcription reaction using QuantiTectRT kit (Qiagen Inc, Valencia, California, USA). Four sets of primers for *CASC8* amplification were designed using Primer3 (http://bioinfo.ut.ee/primer3-0.4.0/primer3/) (Supplementary Table 4). β-actin (Actb) was used as endogenous control. PCR reactions were performed in 25 uL final reaction volume using 20 ng of SCAP cDNA, 1uL of each forward and reverse primers, 0.5 uL Taq DNA polymerase, MgCl2, deoxynucleotide mix, 10 × PCR buffer, and water. Reaction conditions were: 40 cycles at 95 °C (10′), 94 °C (1′), 56 °C (1′), and 72 °C (2′). Products were resolved in agarose gel electrophoresis and imaged using Odyssey Scanner (LI-COR Biosciences, Lincoln, NE).

### Data availability

The datasets generated during and/or analyzed during the current study are available from the corresponding author on reasonable request.

## Electronic supplementary material

Supplemental Information
