# Supplementary material for: Colorectal Cancer-Associated Genes Are Associated with Tooth Agenesis and May Have a Role in Tooth Development
Source: Sci Rep. 2018 Feb 14;8:2979. doi: 10.1038/s41598-018-21368-z (PMC5813178; doi:10.1038/s41598-018-21368-z)
Supplement: Supplementary file 1 — Supplemental Information [file 41598_2018_21368_MOESM1_ESM.doc]

**Colorectal Cancer-Associated Genes Are Associated with Tooth Agenesis and May Have a Role in Tooth Development**

Meredith A. Williams,^1^ Claudia Biguetti,^1,2^ Miguel Romero-Bustillos,^3^ Kanwal Maheshwari,^1^ Nuriye Dinckan,^1,4^ Franco Cavalla,^1,2^ Xiaoming Liu,^5^ Renato Silva,^1,6,7^ Sercan Akyalcin,^8^ Z. Oya Uyguner,^4^ Alexandre R. Vieira,^9^ Brad A. Amendt,^3,10^ Walid D. Fakhouri,^1,7,11^ Ariadne Letra^1,7,11^*

**SUPPLEMENTARY INFORMATION**

**Supplementary Table** **1.** Demographic characteristics of the study population

|  | TA cases  (n=93) | Controls  (n=347) | P-value^b^ |
| --- | --- | --- | --- |
| Males | 29 | 101 | 0.69 |
| Females | 64 | 246 |  |
| Hypodontia | 61 | ---- | ---- |
| Oligodontia | 32 | ---- | ---- |
| Positive family history of cancer | 28 | 39 | 0.00001 |
| *Cancer types ^a^*  *Bladder*  *Brain*  *Breast*  *Colon*  *Other gastrointestinal (esophagus,*  *stomach, intestine)*  *Kidney*  *Leukemia*  *Liver*  *Lung*  *Ovary*  *Pancreas*  *Prostate*  *Skin*  *Thyroid*  *Throat*  *Uterus* | 1  --  4  4  --  1  --  --  --  2  --  4  2  1  --  -- | 1  1  7  1  7  2  2  1  3  --  1  4  5  --  1  3 |  |

^a^ known cancer types reported, no statistical analyses performed due to small numbers

^b^ Chi-square, p≤0.05 indicates statistical difference

**Supplementary Table 2.** Summary of haplotype association results

| **Locus** |  | **Haplotype** | **Frequency (cases)** | **Frequency (controls)** | **P-value^*^** |
| --- | --- | --- | --- | --- | --- |
| *DUSP10* | |  |  |  |  |
| rs6687758|rs6691170 | | GT | 0.1807 | 0.1232 | 0.0443 |
| rs6687758|rs6691170 | | AT | 0.1325 | 0.1938 | 0.0567 |
| rs6687758|rs6691170 | | GG | 0.2479 | 0.0821 | **7.32 x 10^-10^** |
| rs6687758|rs6691170 | | AG | 0.4389 | 0.6009 | *9.20 x 10^-5^* |
| *ATF1* | |  |  |  |  |
| rs7136702|rs11169552 | | TT | 0.1092 | 0.0862 | 0.3390 |
| rs7136702|rs11169552 | | CT | 0.3414 | 0.4019 | 0.1376 |
| rs7136702|rs11169552 | | TC | 0.3194 | 0.1437 | **4.82 x 10^-8^** |
| rs7136702|rs11169552 | | CC | 0.2301 | 0.3683 | *0.0005* |
| *CASC8* | |  |  |  |  |
| rs10505477|rs7014346|rs6983267 | | CAT | 0.0809 | 0.0074 | **4.08 x 10^-9^** |
| rs10505477|rs7014346|rs6983267 | | CGT | 0.3729 | 0.3905 | 0.6643 |
| rs10505477|rs7014346|rs6983267 | | CAG | 0.0917 | 0.0032 | **5 x 10^-13^** |
| rs10505477|rs7014346|rs6983267 | | TAG | 0.2865 | 0.3299 | 0.2626 |
| rs10505477|rs7014346|rs6983267 | | CGG | 0.0449 | 0.0193 | 0.0471 |
| rs10505477|rs7014346|rs6983267 | | TGG | 0.1229 | 0.2496 | *0.0002* |

* Significant association at genome-wide level in bold (if P ≤ 5 x 10^-8^); Positive association under Bonferroni correction in italic (if P ≤ 0.002).

**Supplementary Table** **3.** Results of logistic regression analyses adjusted by positive family history of cancer.

| **Variable** | **Genotype** | **Coefficient** | **Std Error** | **F-test** | **P-Value^a^** |
| --- | --- | --- | --- | --- | --- |
| *ATF1* rs11169552 | CC | 0.129 | 0.128 | 1.0033 | 0.31 |
|  | CT | 0.153 | 0.129 | 1.4056 | 0.23 |
|  | TT | 0.582 | 0.14 | 17.254 | **0.00004** |
| *DUSP10* rs6687758 | AA | 0.118 | 0.172 | 0.4727 | 0.49 |
|  | AG | 0.196 | 0.174 | 1.2796 | 0.26 |
|  | GG | 0.544 | 0.182 | 8.8753 | **0.003** |
| *CASC8* rs10505477 | AA | 0.127 | 0.162 | 0.6145 | 0.43 |
|  | AG | 0.218 | 0.16 | 1.852 | 0.17 |
|  | GG | 0.379 | 0.164 | 5.381 | **0.02** |

^a^ Manzel-Haenzel test.

**Supplementary Table** **4.** Primer sequences for *CASC8* amplification

| **Primer set** | **Forward (5’ - 3’)** | **Reverse (5’ - 3’)** |
| --- | --- | --- |
| 1 | CTTCTGTCCTCAGCGGAAAC | TTGCCGGTAACCTAGATTGG |
| 2 | TTGCACCTGCTATTCCCTCT | GAGGCAGGCAGAAACTGAAC |
| 3 | GAGCCAGAAAATGCAAGGAG | CGACGTTCAAGGTGGCTTAT |
| 4 | AGAGCCAGAAAATGCAAGGA | CGACGTTCAAGGTGGCTTAT |

**
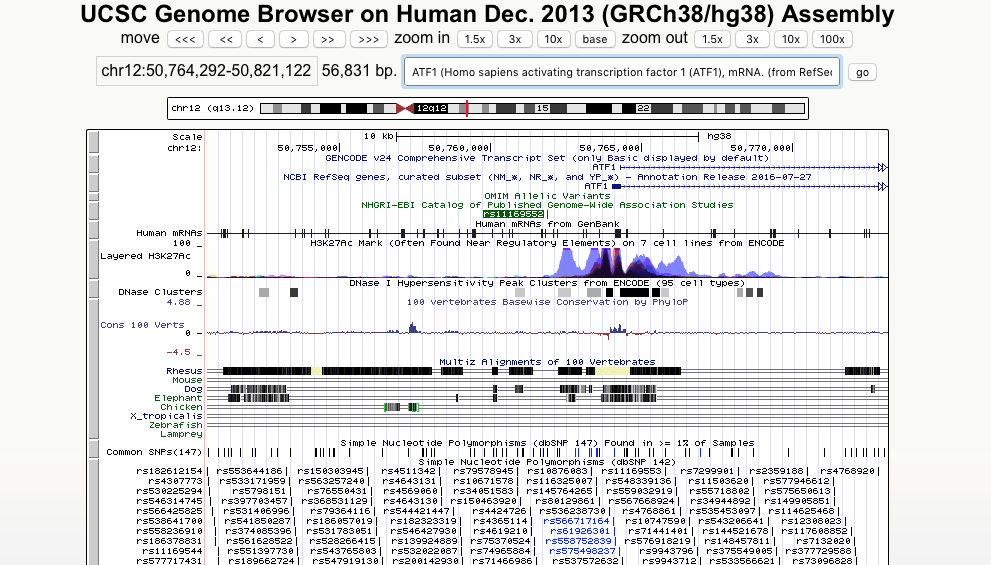
**

**Supplementary Figure 1.** *ATF1* rs11169552 is located in the gene promoter, in a region showing high DNase I hypersensitivity peak clusters (red box) and potential location of regulatory regions, including enhancers, silencers, promoters, insulators, and locus control region. Figure was generated using the UCSC Genome Browser GRCh38/hg38 Assembly (Kent et al. 2002; <https://genome.ucsc.edu/)>.

**
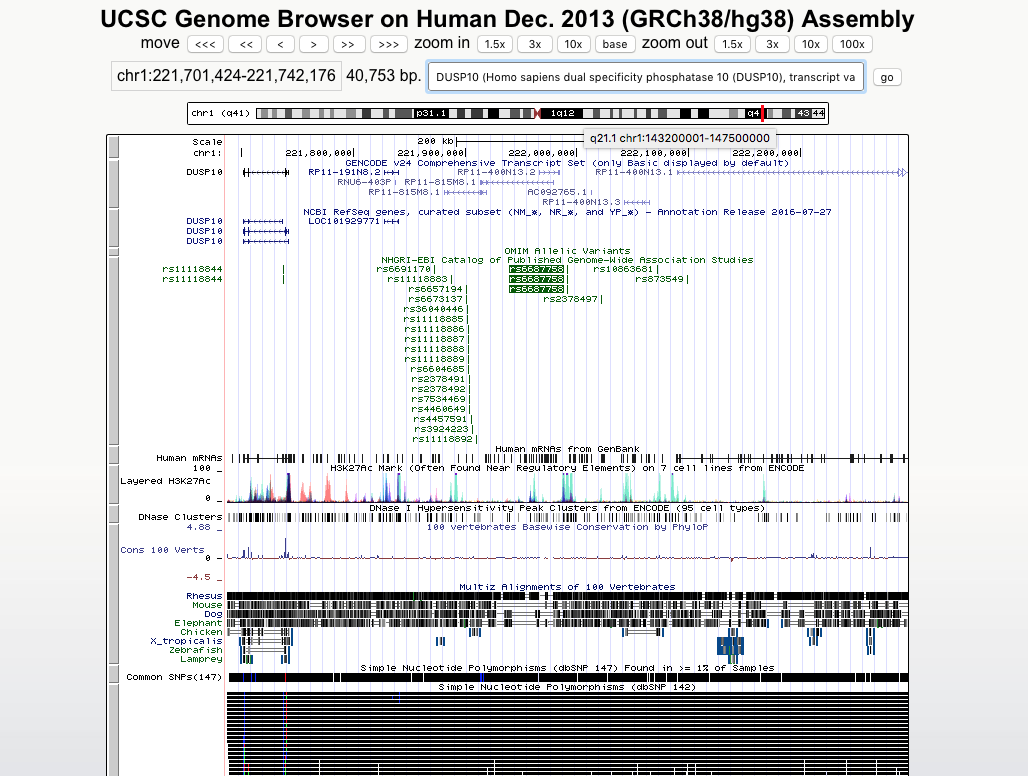
**

**Supplementary Figure 2.** *DUSP10* rs6687758 is located downstream of the gene, and falls in a region showing high DNase I hypersensitivity peak clusters (red box) and potential location of regulatory regions. Figure was generated using the UCSC Genome Browser GRCh38/hg38 Assembly (Kent et al. 2002; <https://genome.ucsc.edu/)>.

**
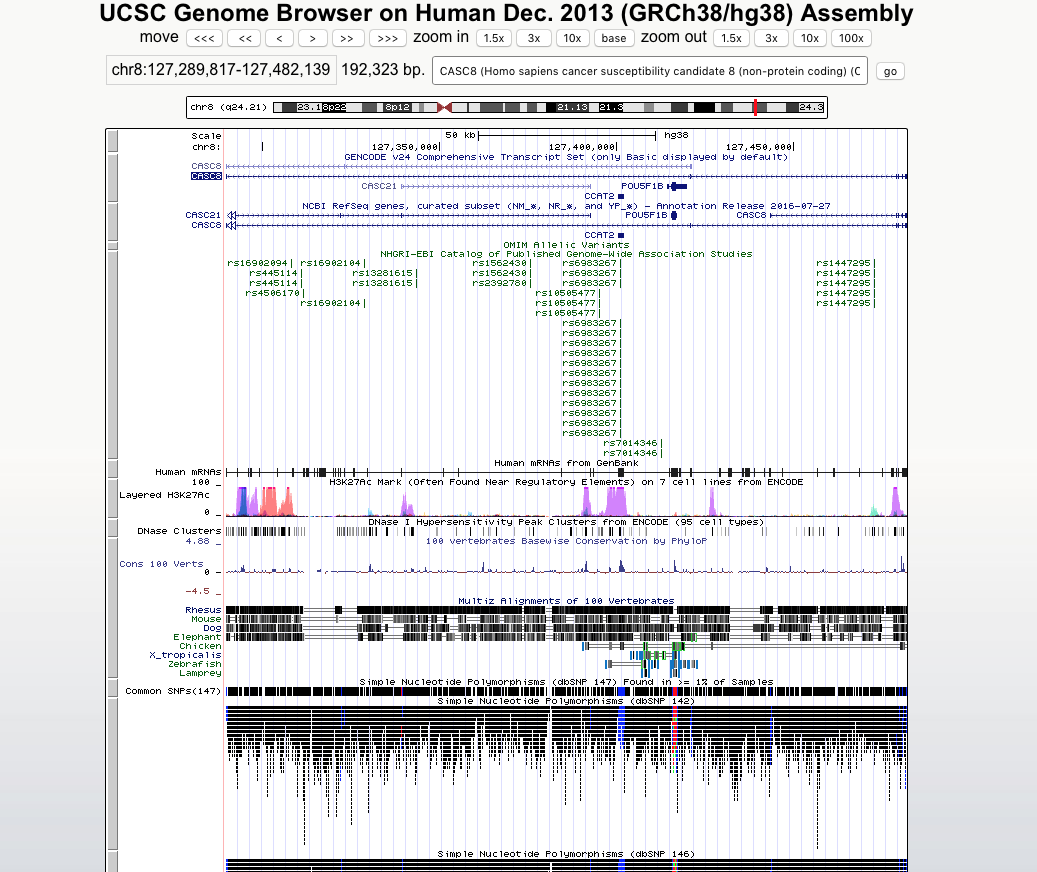
**

**Supplementary Figure 3.** *CASC8* rs10505477 is located in a region of enriched H3K27Ac histone marks (red box), which are found nearby regulatory regions. Figure was generated using the UCSC Genome Browser GRCh38/hg38 Assembly (Kent et al. 2002; <https://genome.ucsc.edu/)>.


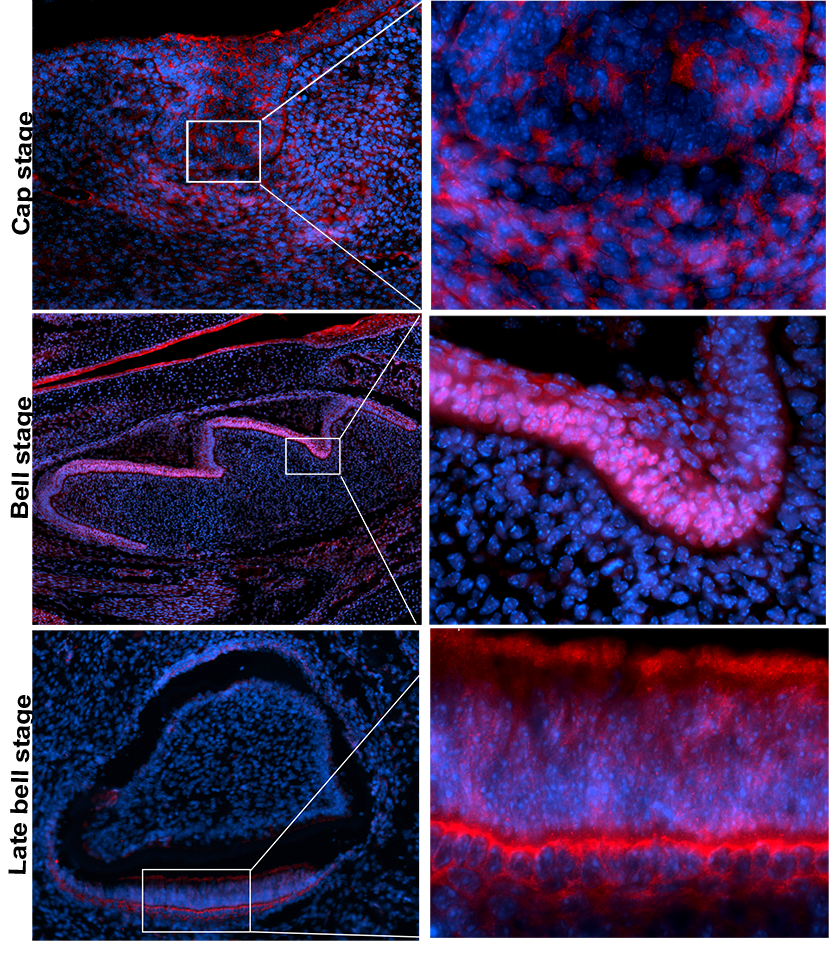


**Supplementary Figure 4.** ATF1 expression at cap, bell and late bell stages of tooth development


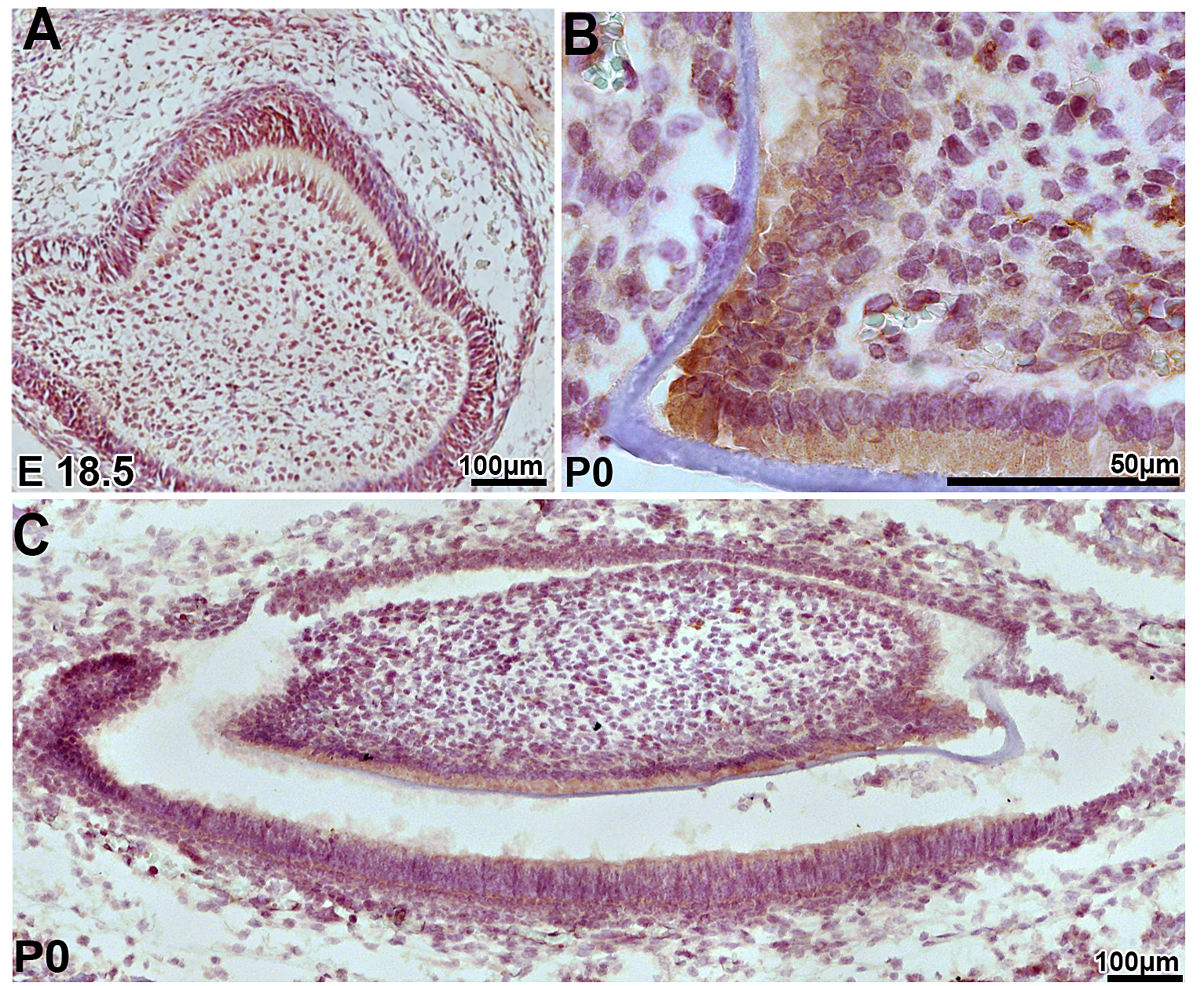


**Supplementary Figure 5.** DUSP10 expression at bell and late bell stages of tooth development

**
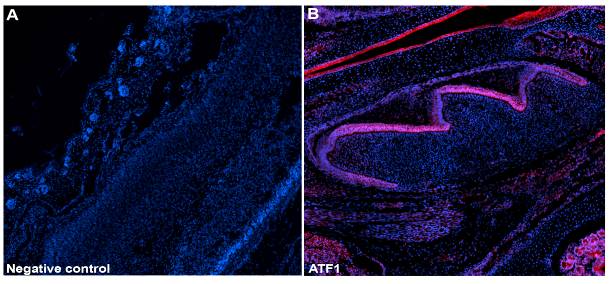
**

**A**

**B**

**
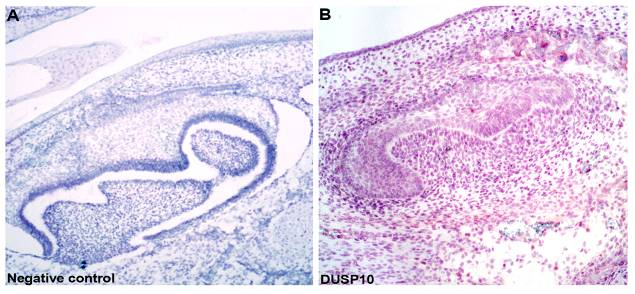
**

**C**

**D**

**Supplementary Figure 6.** Negative control sections and positive immune staining sections for ATF1 and DUSP10. (A) Negative control section for ATF1 with secondary antibody goat anti-rabbit Alexa 555 and DAPI. (B) Immune positive staining for ATF1 (Alexa 555) and DAPI. (C) Negative control section for DUSP10 with secondary antibody horse anti-rabbit IgG and VECTOR NovaREDPeroxidasechromogen. (D) Immune positive staining for DUSP10, with VECTOR NovaRED chromogen and counterstaining with Mayer’s Hematoxylin.
